# miR-135a Reduces Osteosarcoma Pulmonary Metastasis by Targeting Both BMI1 and KLF4

**DOI:** 10.3389/fonc.2021.620295

**Published:** 2021-03-22

**Authors:** Chenglong Chen, Xingjia Mao, Caitong Cheng, Yurui Jiao, Yi Zhou, Tingting Ren, Zhuangzhuang Wu, Zhi Lv, Xiaojuan Sun, Wei Guo

**Affiliations:** ^1^ Musculoskeletal Tumor Center, Peking University People’s Hospital, Beijing, China; ^2^ Beijing Key Laboratory of Musculoskeletal Tumor, Peking University People’s Hospital, Beijing, China; ^3^ Shanxi Key Laboratory of Bone and Soft Tissue Injury Repair, Second Hospital of Shanxi Medical University, Taiyuan, China; ^4^ Endocrinology Research Center, Xiangya Hospital Central South University, Changsha, China; ^5^ Department of Plastic and Aesthetic Surgery, Nanfang Hospital of Southern Medical University, Guangzhou, China

**Keywords:** osteosarcoma, metastasis, KLF4, BMI1, microRNA

## Abstract

Because of the modest response rate after surgery and chemotherapy, treatment of osteosarcoma (OS) remains challenging due to tumor recurrence and metastasis. miR-135a has been reported to act as an anticarcinogenic regulator of several cancers. However, its expression and function in osteosarcoma remain largely unknown. Here, we reported that abridged miR-135a expression in OS cells and tissues, and its expression is inversely correlated with the expression of BMI1 and KLF4, which are described as oncogenes in several cancers. Ectopic expression of miR-135a inhibited cell invasion and expression of BMI1 and KLF4 in OS cells. *In vivo* investigation confirmed that miR-135a acts as a tumor suppressor in OS to inhibit tumor growth and lung metastasis in xenograft nude mice. BMI1 and KLF4 were revealed to be direct targets of miR-135a, and miR-135a had a similar effect as the combination of si-BMI1 and si-KLF4 on inhibiting tumor progression and the expression of BMI1 and KLF4 *in vivo*. Altogether, our results demonstrate that the targeting of BMI1/KLF4 with miR-135a may provide an applicable strategy for exploring novel therapeutic approaches for OS.

## Introduction

Human osteosarcoma (OS), accounting for approximately 56% of bone sarcomas, is the most common malignant bone tumor and typically affects children, adolescents, and young adults with a median age of 16 years (1, 2). Clinically, OS is often diagnosed with lung metastasis at late stages due to its high proclivity for the local invasion and preferential metastasis to pulmonary parenchyma, in addition, recurrent disease is reported to be observed in 30%-50% of the population (2–5). The current standard care for OS consists of extensive surgical resection, neoadjuvant chemotherapy, and adjuvant chemotherapy (6). Although several anticarcinogens have been applied clinically, the prognosis for metastatic and recurrent OS has remained stagnant over the last few decades (7). Around 68% of localized OS patients survive for five years or longer (8), but unfortunately, 20–30% suffer from metastatic or recurrent tumors (9). Given the barriers to conventional treatment methods, there is a crying need to establish novel therapeutic strategies that may improve the overall survival of OS.

B cell-specific Moloney murine leukemia virus integration site 1 (BMI1) is the core component of the PRC1 complex and is reported as an oncogene able to induce cell transformation and self-renewal and promote tumor growth (10, 11). It has been reported that BMI-1 also plays a critical role in OS cell migration, metastasis (11), and chemosensitivity toward cisplatin-induced apoptosis (12, 13). Krüppel-like factor 4 (KLF4), also referred to as gut-enriched Krüppel-like factor and GKLF, belongs to the SP/KLF family that are characterized by three zinc finger motifs and regulates various biological processes, including differentiation, cell cycle progression, proliferation, cellular migration, and stem cell renewal (14–17). KLF4 was found to increase and act as an oncogene in several cancers, including OS (16, 17). The extracellular matrix (ECM) hosts some structural molecules and enzymes in part to create cellular microenvironments or niches (18, 19). Failing to regulate ECM remodeling results in the development of pathological processes, such as connective tissue disorders, cancers, as well as metastasis (20, 21). Matrix metalloproteinases (MMPs) are reported to involved in ECM degradation (22), and our previous study showed that some MMPs play vital roles in the invasion and metastasis of OS cells (8). Some MMPs function as downstream nodes of the BMI1 and KLF4 pathways, which suggests that these two molecules may also promote tumors through MMPs (23, 24).

MicroRNAs (miRNAs) are a family of small, noncoding RNA molecules that regulate gene expression at the posttranscriptional level through either mRNA cleavage or translational repression through direct binding to the 3′ untranslated region (3′-UTR) (25, 26). Our previous studies indicated that upregulation of some miRNAs could intensely restrain the growth, invasion, and metastasis in OS xenograft tumors and attenuate angiogenesis (8, 27). We also found that the level of miRNA-135 (miR-135a-5p) was reduced in OS tissues from patients in whom BMI1, KLF4, and MMP expression was upregulated. In addition, these molecules were theoretically predicted to be direct targets of miR-135a in a bioinformatics database, which was confirmed by our study. In the present study, we explored the effects of miR-135a on targeting of BMI1, KLF4, and MMPs to suppress OS cell proliferation, invasion, and lung metastasis and we also compared the therapeutic effects of miR-135a, the combination of anti-BMI1 and anti-KLF4, and PTC-209 *in vitro* and *in vivo*. Consequently, miR-135a, as a novel cancer suppressor gene, may have implications for the treatment of OS.

## Materials and Methods

### Clinical Tissue Specimen

The biopsies from 10 OS patients were acquired during the diagnostic process at Second Hospital of Shanxi Medical University (Taiyuan, China) and then subjected to RT-qPCR. The paraffin-embedded samples from 10 OS patients and non-malignant cartilage from 3 patients for knee replacement surgery were subjected to immunohistochemistry. Informed consent was obtained from all patients, and the protocols in this study were approved by the Ethics Committee of Shanxi Medical University (2017LL077).

### Cell Lines and Culture

The human OS cell lines (MG-63 and Saos-2) were obtained from ATCC and cultured at 37°C in a humidified incubator with 5% CO^2^ in RPMI-1640 and Dulbecco’s modified Eagle’s medium (DMEM) supplemented with 10% fetal bovine serum (FBS), plus antibiotics. Osteoblast-like cells were isolated from cartilage tissue and cultured in α-MEM as described in a previous study (28).

### Cell Transfection, Transduction, and Compounds

Saos-2 cells were cultured overnight to reach 60% confluence and transfected with siRNAs: si-BMI1, si-KLF4, a combination of si-BMI1 and si-KLF4 (si-B+K), or control siRNA (Genepharma, Shanghai, China) at a final concentration of 20 nM in OPTI-MEM for 48 h using a Lipofectamine 3000 kit (Thermo Fisher Scientific) following the instructions. Similarly, the cells were transduced with lentiviral miR-135a (LV-miR-135a; Genechem) or lentiviral control miR-NC in the 5 μg/ml Polybrene for 24 h and treated with 3 μg/ml of puromycin for 3 days. miR-135a expression in generated cell clones was tested for stable expression. Correspondingly, the cells were treated with PTC-209 (an inhibitor of BMI1, purchased from MedChemExpress company, Monmouth Junction, NJ, USA) at a final concentration of 10 μM diluted in DMSO or their DMSO control for 2 days. The cells were grown to 80% confluence after subculture in complete medium for further experiments.

### RNA Isolation and qRT-PCR

Total RNA and miRNAs were extracted from Osteoblast-like cells and OS cell lines and then transfected cells with a TRIzol reagent (Thermo Fisher Scientific) and the miRNeasy Mini Kit (Qiagen, MD, USA) following the protocol provided by the manufacturer. qRT-PCR was utilized to measure the relative expressions of target gene to the control 18S rRNA or U6 transcripts with a IQ5 Multicolour Real-Time PCR Detection system (Bio-Rad Laboratories, CA, USA). The conditions of the PCRs for miR-135a were as described in our recent publication (8). The primers sequences of U6 were 5′-CTCGCTTCGGCAGCACA-3′ and 5′-AACGCTTCACGAATTTGCGT-3′. The primers sequences of miR-135a-5p were 5′AACCCTGCTCGCAGTATTTGAG-3′ and 5′-GCGGCAGTATGGCTTTTTATTCC-3′. The data were normalized to the controls and analyzed by using the comparative CT method (2^−ΔΔCt^) (29).

### Transwell Invasion Assay

Saos-2 cells were divided into 8 groups: control miR-NC (NC), LV-miR-135a (miR-135), control siRNA (Control), si-BMI1, si-KLF4, combined si-BMI1 and si-KLF4 (si-B+K), DMSO, and PTC-209 at a final concentration of 10 μM (PTC-209). The invasion assay was performed using chambers of 24‐well plates coated with Matrigel matrix on the 8−μm pore size membrane of the upper chamber. A total of 180 μl of Matrigel Matrix Reduced (Corning, NY, US) diluted 1:3 with serum-free medium was used to coat 24-well inserts and incubated for 2 h. Two hundred microliters of Saos-2 cell suspensions were then added into the upper chambers at a density of 1×10^6^ cells/mL in RPMI 1640 medium containing 1% FBS. The basolateral chambers were filled with 600 μL RPMI 1640 medium with 10% FBS. After incubation for 72 h with 5% CO2 at 37°C, the cells remaining on the upper membrane were removed by cotton swabs, and cells that invaded across the membrane were washed twice and fixed with 4% paraformaldehyde for 15 min and stained with 0.5% crystal violet for 15 min. The invasive cell numbers were calculated in 5 images per well.

### 5-Ethynyl-20-Deoxyuridine (EdU) Assay

For the EdU assay, si-KLF4/BMI1, control siRNA, LV-miR-135a or the corresponding NC were transfected, and PTC-209 or DMSO was added to Saos-2 cells. Then, the cells were cultured in the supplement of EdU for 8 h, and the cells were fixed with 4% paraformaldehyde for 15 min. Then, 0.5% Triton™ X−100 was employed for 20 min to permeabilize the nuclear membrane, and used 5% normal serum for blocking at 25°C for 1 h. Finally, cells were stained with a Cell-Light™ EdU Apollo^®^488 *in vitro* Imaging Kit (Life Technologies, New York, USA). Staining was observed under a Pannoramic MIDI digital slide scanner (3DHISTECH, Ltd. Budapest, Hungary). The level of proliferation was represented as a percentage of the EdU-positive cells to the total cells.

### Immunofluorescence Assay

A total of 3×10^5^ cells were seeded on glass coverslips overnight and then fixed for 15 min with 4% paraformaldehyde. Blocking buffer (5% normal serum and 0.5% Triton™ X−100) was used for blocking for 1 h. Next, coverslips were incubated with specific primary antibodies overnight at 4°C. The primary antibodies were as follows: KLF4 (#BS90773, BioWorld, Nanjing, China), BMI1 (#10832-1-AP, Proteintech, Rosemont, IL, USA), MMP2 (#bs-0412R, Bioss, London, UK), MMP9 (#bs-4593R, Bioss, London, UK), Ki67 (#ab15580, Cambridge, UK). The cells were then incubated in fluorochrome−conjugated secondary antibody and stained with DAPI (Cell Signalling Technology, Danvers, MA, USA), and slides were examined under a slide scanner.

### In Vivo Growth and Bioimaging Analysis

Female BALB/c nude (n=50; 5 weeks old) mice were obtained from Charles River Laboratory (Beijing, China), implanted subcutaneously with 1×10^6^/200 μL of Saos-2 cells mixed with 200 μL Matrigel (Corning, NY, USA) into the back flank of each mouse and maintained in a SPF “barrier” facility. Two weeks after implantation, the mice were randomly divided into 5 groups (n=10 in each group) and injected intratumorally with 5 nmol combination of KLF4 and BMI1 siRNA (Combination treatment/si-B+K, RIBOBIO, Ltd. Guangzhou, China), cholesterol-conjugated 2′-O-methyl-modified miR-135a mimics (miR-135 mimic) or their controls (NC), and 30 mg/kg PTC-209 or 8% DMSO once every 2 days for 2 weeks. The dynamic growth tumors were monitored every day. At week 2 postinoculation, bioimaging for MMP activity *in vivo* was detected by MMPSense 680 (PerkinElmer) and performed with fluorescence molecular tomography (FMT) (PerkinElmer, Waltham, USA). The mice were sacrificed, and their tumors and lungs were dissected. Tumors were measured using callipers, and the volumes were calculated according to our pervious study (8). Weight was measured after excision. Lung samples were stored in 10% formalin for HE staining. Tumor samples were stored in 10% formalin, RNAlater, or RIPA buffer for later use.

### Hematoxylin and Eosin (HE) Staining

The resection and farther processing of mouse lung samples for the HE staining were performed as described in our previous study (8). After HE staining, the sections were analyzed with a Pannoramic MIDI digital slide scanner. Each slide was examined for metastases, and metastatic burden was evaluated as the number of nodules per lung.

### Immunohistochemistry

The xenograft tumor tissues, tumor tissues from OS patients, and nonmalignant bone tissues were fixed with 10% formalin for 2 days, and the tumor sections (5 μm) were dewaxed, rehydrated, and blocked using a standard immunohistochemical staining procedure as described in our previous studies (8, 27). The sections were stained with anti-KLF4/BMI1/MMP2/MMP9 at 4°C overnight. The staining intensity was analyzed by immunoreactive score (IRS) system (8) using a slide scanner and software (3DHISTECH, Ltd. Budapest, Hungary).

### Western Blotting

The mouse xenograft tumor tissues were homogenized in the RIPA lysis buffer with PMSF, phosphatase and protease inhibitors (Keygen, Nanjing, China). The concentrations of total proteins in each sample were detected by bicinchoninic acid (BCA). Protein samples of each loading was separated in 10% sodium dodecyl sulfate-polyacrylamide gel electrophoresis (SDS/PAGE) gels and transferred onto polyvinylidene difluoride membrane (Millipore, Danvers, MA, USA). The polyvinylidene difluoride membranes were blotted with anti-KLF4, anti-BMI1, anti-MMP-2, anti-MMP-9, and anti-β-actin (Bioss, London, UK). The immunoblots on the membranes were developed with the enhanced chemiluminescence reagent. The band intensity was determined by densitometric analysis using IMAGEJ software for relative quantification of protein levels by normalized with β-actin in the tumor.

### Dual-Luciferase Reporter

A total of 2×10^5^ Saos-2 cells were inoculated into 24-well plates per well. When the confluence of cells reached 80%, the cells were transiently transfected with 10 nM miR-135a mimic or miR-NC and cotransfected with 0.25 μg per/well of plasmids containing 3′-UTR of BMI1 or KLF4 or their mutant (GenePharma, Shanghai, China) using Lipofectamine 3000 (Invitrogen) for 2 days. Total cell protein was extracted using RIPA buffer and quantified using the bicinchoninic acid method. The luciferase activity in each well was measured in a GLOMAX 20/20 luminometer (Promega) using the Dual-Luciferase Reporter Assay System (Promega Corp, Madison, USA) following the manufacturer’s instructions. Luciferase values were normalized for transfection efficiency (firefly/Renilla ratios).

### Kaplan-Meier Survival Analyses

Kaplan-Meier survival analyses was performed on R2: Genomics Analysis and Visualization Platform (http://r2.amc.nl). Gene expression in single gene (BMI1 or KLF4) was selected on Mixed Osteosarcoma Dataset and cut-off modus of scan was selected for overall survival analysis.

### Statistical Analysis Assay

All data are presented as the mean ± SEM, and the significance of differences among groups were analyzed by ANOVA test, and the difference in unpaired groups was analyzed using Student’s *t*-test. Using Mann–Whitney U test for comparisons between two groups, and in multiple groups the Kruskal-Wallis-H test was used when data did not coincide with a normal distribution. Repeated measures ANOVA was used for repeated measurement data. Statistical significance was set at *p* < 0.05 and the analyses were performed using SPSS (version 21.0, SPSS Inc., Chicago, USA).

## Results

### Aberrant Expression of miR-135a, BMI1, and KLF4 in Human OS Tissues and Cells

**Figure 1:**
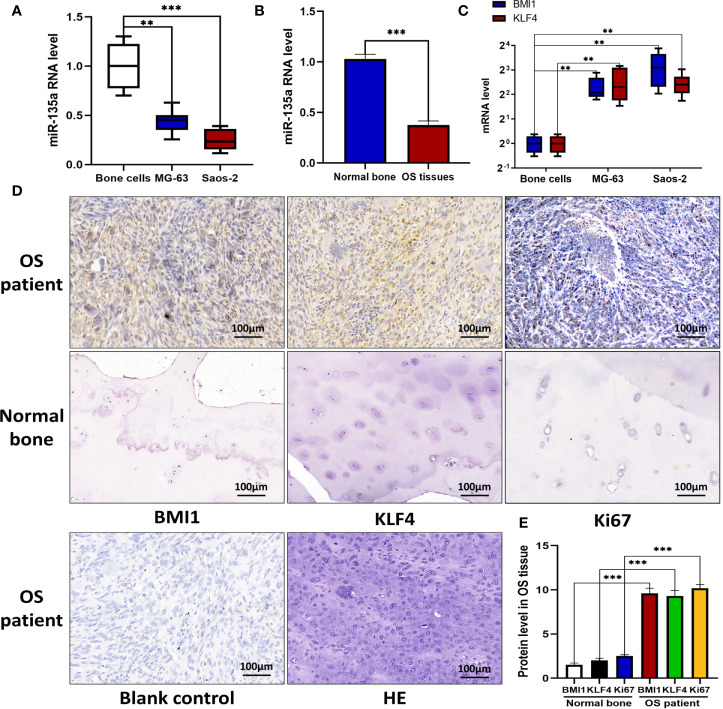
Downregulation of miR-135a and upregulation of BMI1 and KLF4 in OS cell lines and tissues. **(A)** RT-qPCR. OS cell lines Saos-2 and MG-63, and primary normal bone cells (osteoblasts) were grown and then subjected to RT-qPCR analysis. The results showed that endogenous miR-135a expression was decreased in Saos-2 and MG63 cells when compared to osteoblasts (**p < 0.01; ***p < 0.001; n=3). **(B)** RT-qPCR. Levels of endogenous miR-135a expression was decreased in OS tissues when compared to normal bone (***p < 0.001; n=3). **(C)** RT-qPCR. Levels of BMI1 and KLF4 mRNA were higher in Saos-2 and MG63 cell lines compared with osteoblasts (**p < 0.01; n=3). **(D)** Immunohistochemistry. Human OS and normal bone tissues were collected and subjected to immunohistochemical analysis. Scale bar, 100 μm. **(E)** The graph represents quantified data of the immunohistochemistry (***p < 0.001; n = 10). All the data from qPCR were expressed as the mean ± SEM.

In this study, we first assessed the expression of miR-135a in OS cell lines, primary human normal bone cells, OS tissue, and normal bone tissue using qRT-PCR and we found that the miR-135 expression in Saos-2 and MG-63 cells or OS tissue were lower than those in nonmalignant bone cells or tissue (**p < 0.01; ***p < 0.001; **Figures 1A, B**). We further examined expression level of BMI1 and KLF4 in OS cell lines and normal bone cells using qRT-PCR, and expression level of BMI1, KLF4, and Ki67 in human OS samples using immunohistochemistry also found that the BMI1 and KLF4 expressions were significantly higher in OS cell lines than in normal bone cells (**p < 0.01; **Figure 1C**) or higher in human OS samples than in nonmalignant bone samples (***p < 0.001; **Figure 1D**).

### miR-135a, Combination Treatment, and PTC-209 Suppress OS Cell Invasion and Proliferation

**Figure 2:**
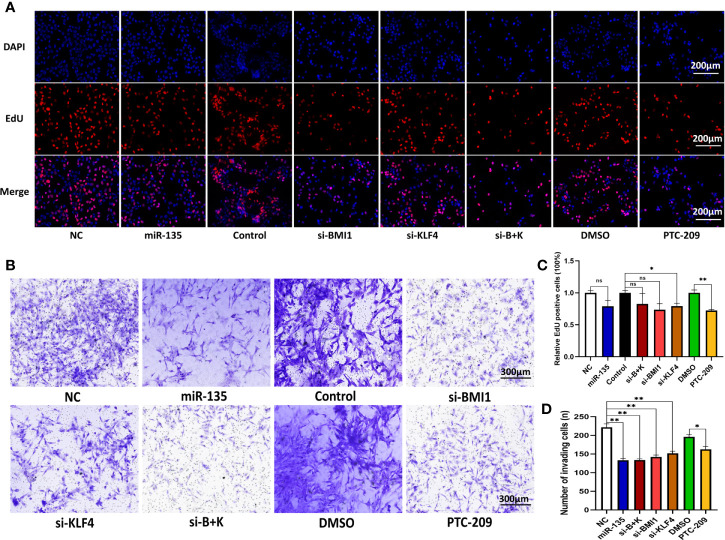
Downregulating of KLF4 and using PTC-209 suppress the OS cell proliferation; miR-135a, si-BMI1, si-KLF4, combination therapy, and PTC-209 suppress the OS cell invasion. **(A)** Representative pictures from tumor cell EdU assay. Saos-2 cells were grown and transfected with Lv-miR-135a or negative controls, si-BMI1/KLF4 or their control, and treatment with PTC-209 or DMSO, and then subjected to the EdU assay. Scale bar, 200 μm. **(B)** Representative pictures from transwell invasion assay. The Saos-2 cells with same treatments as above were subjected to the transwell assay. Scale bar, 300 μm. **(C)** The graph is the quantified data of the EdU assay (ns *p* > 0.05; *p < 0.05; **P < 0.01; n = 3). **(D)** The graph is the quantified data of the transwell invasion assay (*p < 0.05; **p < 0.01; n = 3). The data were expressed as the mean ± SEM. NC (Lentivirus negative control), miR-135 (Lentivirus overexpressed miR-135a), Control (siRNA negative control), si-BMI1 (group interfered with siRNA-BMI1), KLF4 (group interfered with siRNA-KLF4), si-B+K (group interfered with siRNA-BMI1 and siRNA-KLF4). ns, not significant.

We next assessed and compared the role of miR-135a, si-BMI1, si-KLF4, combined si-BMI1 and si-KLF4 (combination therapy), and PTC-209 on OS cell proliferation and motility using EdU assays and Transwell invasion assays (**Figures 2A, B**). Our data presented that overexpression of miR-135a decreased the invasion capability of OS cells (*p < 0.05; **p < 0.01; **Figures 2B, D**). Similarly, we observed that si-BMI1, si-KLF4, combination therapy, and PTC-209 all significantly inhibited tumor cell invasion compared with each control group (*p < 0.05; **p < 0.01; **Figures 2B, D**). Furthermore, among the five groups, miR-135a was significantly superior to si-KLF4 in inhibiting cell invasion (*p < 0.05), and PTC-209 was inferior to the other reagents (*p < 0.05). Using the EdU assay, we observed that si-KLF4 and PTC-209 significantly inhibited tumor cell proliferation compared with each control group (*p < 0.05; **p < 0.01; **Figures 2A, C**). However, there were no significant differences in proliferation capacity among the five treatment groups (*p*>0.05).

### miR-135a, Combination Therapy, and PTC-209 Inhibition of Xenograft Growth and Pulmonary Metastasis

**Figure 3:**
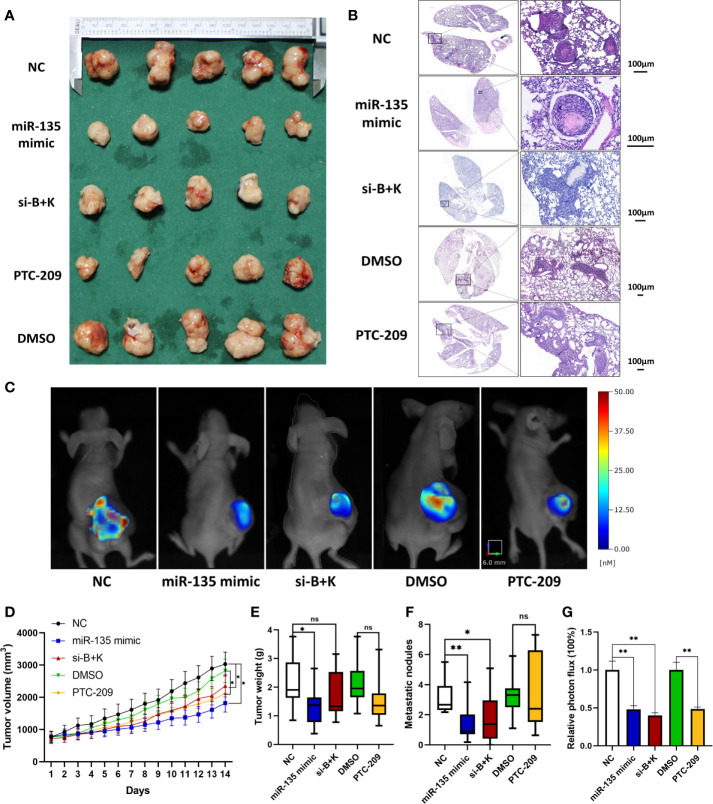
miR-135a, combination therapy, and PTC-209 suppression of mouse OS cell xenograft growth; miR-135a and combination therapy suppression of lung metastasis *in vivo*. **(A)** Xenograft photographs. Saos-2 cells were grown and injected into nude mice, and then treat mice with miR-135 mimic, si-B+K, negative control, PTC-209, and DMSO respectively. After 2 weeks, the mice were sacrificed and the xenografts were photographed. **(B)** HE staining of mouse lung tissues. The mice were sacrificed and the lungs were resected and subjected to tissue processing, embedding, sectioning, and HE staining to quantify tumor cell lung metastasis. Scale bar, 100 μm. **(C)** Bioimages. Before sacrificed, mice were subjected to bioimaging of the MMPs activity in xenograft tumors with the fluorescence molecular tomography using MMPSense 680. **(D)** Growth curves of mouse OS cell xenografts. (*p < 0.05; **p < 0.01; n = 10). The significance of differences among groups were analyzed by ANOVA test and the effects were corrected using the Greenhouse–Geisser method in tumor volume because the data did not meet the hypothesis of sphericity (Mauchly’s test statistic W=0.000, *p < *0.001). **(E)** Weight of mouse OS cell xenografts. (*p < 0.05; ns *p* >0.05; n = 10). **(F)** The graph of the quantified data of metastatic nodules. (ns *p* > 0.05; *p < 0.05; **p < 0.01; n = 10). **(G)** The graph of the quantified data of MMPs activity in xenograft tumors. (**p < 0.01; n = 5). ns, not significant.

We tested the miR-135a levels in the control and miR-135 groups (group transfected with miR135a mimics) of xenograft models by using qRT-PCR and confirmed the overexpression of miR-135a in the miR-135 group (***p < 0.001; n=9; **Supplementary Figure 1**). We further examined the effect of miR-135a, combination treatment, and PTC-209 *in vivo* using a mouse tumor cell xenograft model. Our data showed that the xenograft volumes in the therapy groups were smaller than those in each control group. The time difference was statistically significant (**p < 0.01), and the time * group differences were significantly different, with a significant interaction effect within groups * time (*p < 0.05), and the decreased amplitude in miR-135a group was greater than that in other two treatment groups (*p < 0.05; **Figures 3A, D**). The data showed the xenograft weight in miR-135a group was lesser than that in the control NC group(*p*<0.05; **Figure 3E**), but no significant differences in xenograft weights between other treatment groups and each control group or among the miR-135a, combination therapy, and PTC-209 groups (*p*>0.05; **Figure 3E**). The miR-135a, combination therapy, and PTC-209 groups all reduced MMP probe activation and retention, as shown by the fluorescence images of fluorescence molecular tomography (FMT) and the quantification of the intensity of the probe fluorescence signal level in the xenografts, indicating that MMP enzyme activity was lower in the treatment groups than in the control groups (**p < 0.01; **Figures 3C, G**), but there was no significant difference among the three treatment groups (*p*>0.05). Furthermore, miR-135a and combination therapy also inhibited the number of metastatic nodules in the lung (*p < 0.05; **p < 0.01; **Figures 3B, F**).

### miR-135a, si-BMI1, si-KLF4, Combination Therapy, and PTC-209 Inhibited BMI1, KLF4, and MMP Expression in OS Cells

**Figure 4:**
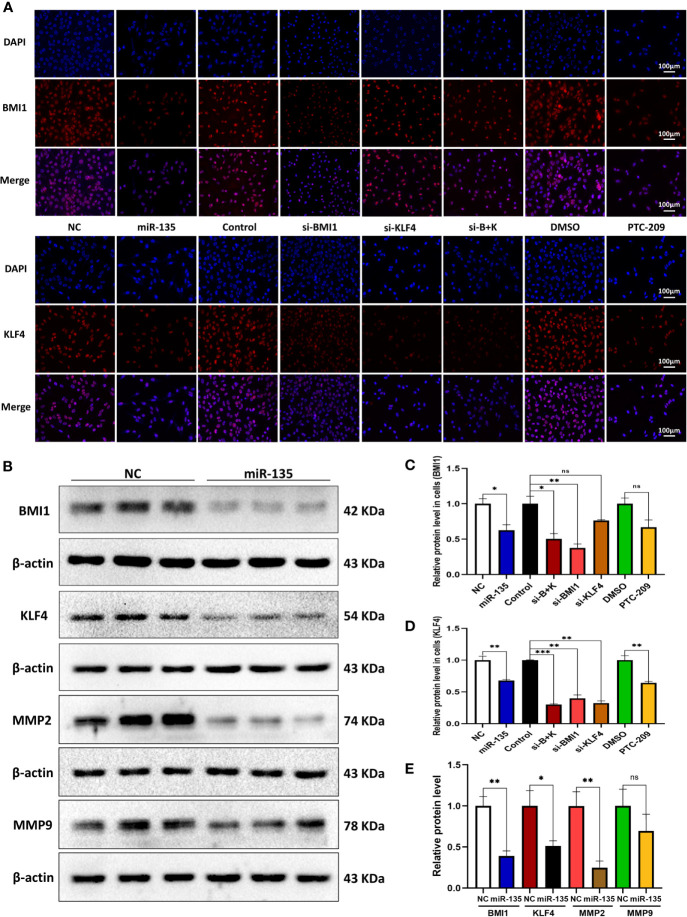
miR-135a, si-BMI1, si-KLF4, combination treatment, and PTC-209 inhibit BMI1 and KLF4 expression in OS cells. **(A)** Representative pictures from tumor cell immunofluorescence assay. The Saos-2 cells with same treatments as above were subjected to the immunofluorescence assay. Scale bar, 100 μm. **(B)** Western blot. Saos-2 cells were grown and transfected with Lv-miR-135a or negative controls and then subjected to the protein extraction and western blot. **(C, D)** The graphs were the quantified data of immunofluorescence staining of BMI1 and KLF4. (ns *p* > 0.05; *p < 0.05; **p < 0.01; n = 3). Quantitation was performed by analyzing the positive cells in relation to DAPI. **(E)** The graph is the quantified data of the western blot. (ns *p* > 0.05; *p < 0.05; **p < 0.01; n = 3). The data were expressed as the mean ± SEM. NC (Lentivirus negative control), miR-135 (Lentivirus overexpressed miR-135a), Control (siRNA negative control), si-BMI1 (group interfered with siRNA-BMI1), KLF4 (group interfered with siRNA-KLF4), si-B+K (group interfered with siRNA-BMI1 and siRNA-KLF4). ns, not significant.

We explored the effect of five approaches (miR-135a, si-BMI1, si-KLF4, combination therapy, and PTC-209) on the expression of BMI1 and KLF4 in OS cells. Immunofluorescence revealed that the approaches all reduced the expression levels of BMI1 and KLF4 in OS cells (*p < 0.05; ** *p* < 0.01; ***p < 0.001; **Figures 4A, C, D**). For BMI1 expression, the si-BMI1 and combination therapy groups showed a greater effect on BMI1 protein reduction than the si-KLF4 group (*p < 0.05); the si-BMI1 and PTC-209 groups showed more decreased protein expression than the miR-135a group (*p < 0.05). For KLF4 expression, the si-BMI1, si-KLF4, and combination therapy groups showed more decreased protein expression than the miR-135a and PTC-209 groups (*p < 0.05). The Western blot results confirmed that miR-135a inhibited BMI1, KLF4, and MMP2 expression in OS cells (*p < 0.05; **p < 0.01; **Figures 4B, E**).

### miR-135a, Combination Therapy, and PTC-209 Suppressed BMI1, KLF4, and MMP9 Expression in Mouse OS Cell Xenografts

**Figure 5:**
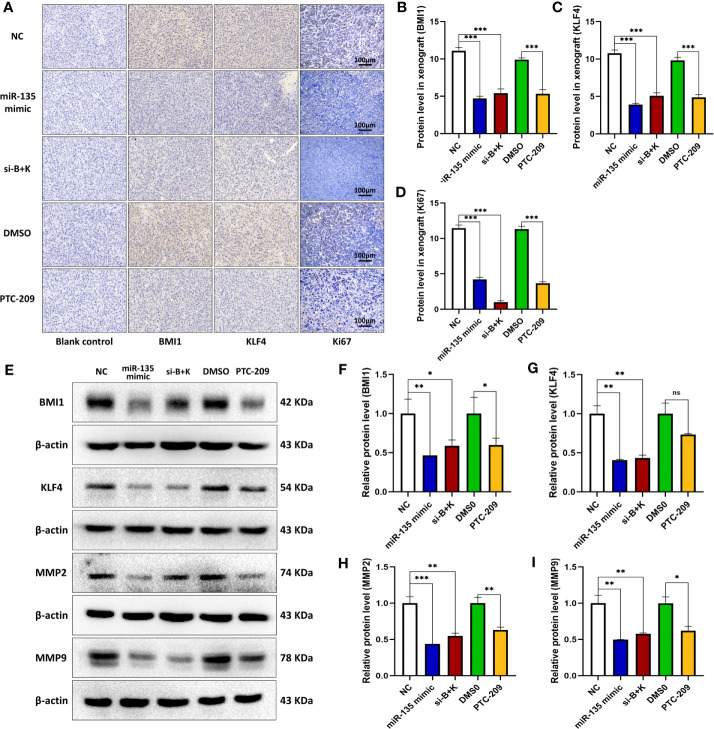
miR-135a, combination therapy, and PTC-209 suppression of BMI1 and KLF4 in mouse OS cell xenografts. **(A)** Immunohistochemistry. The mouse OS cell xenografts were resected from the nude mice and subjected to tissue processing, embedding, sectioning, and immunohistochemistry. Scale bar, 100 μm. **(B–D)** The graph of the quantified data of immunohistochemical staining of BMI1, KLF4, and Ki67. (***p < 0.001; n = 10). **(E)** Western blot. The mouse OS cell xenografts were resected from the nude mice and subjected to protein extraction and western blot. **(E–I)** The graph of the quantified data of the western blots (ns *p*>0.05; *p <0.05; **p < 0.01; ***p < 0.001; n = 3). The data were expressed as the mean ± SEM. ns, not significant.

We further tested the effect of three approaches (miR-135 mimic, si-B+K, and PTC-209) on BMI1, KLF4, and Ki67 expressions in mouse OS xenografts. Immunohistochemistry revealed that the three approaches all suppressed BMI1, KLF4, and Ki67 expression *in vivo* (***p < 0.001; **Figures 5A–D**). For KLF4 expression, the miR-135 mimic and si-B+K groups showed more decreased protein expression than the PTC-209 group (*p < 0.05). We used parallel measure to detect BMI1, KLF4, MMP2, and MMP9 expressions using Western blot and found a similar decrease of BMI1, MMP2, and MMP9 in each group (*p < 0.05; **p < 0.01; ***p < 0.001; **Figures 5E, G–I**), and there was no significant difference among treatment groups (*p* > 0.05). miR-135 mimic and si-B+K both suppressed BMI1, KLF4, MMP2, and MMP9 expression (**p < 0.01; ***p < 0.001; **Figures 5D–I**).

### Positive BMI1 and KLF4 Expression Are Associated With OS Metastasis, and Predicted a Short Survival Time

**Figure 6:**
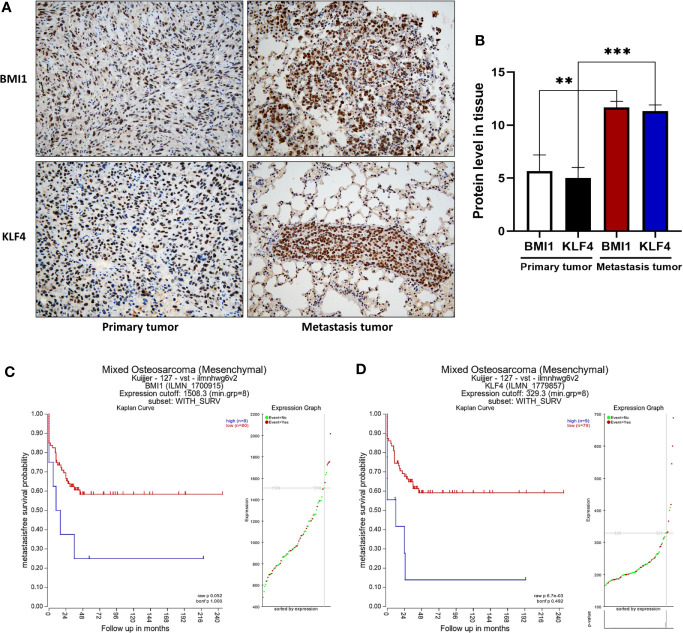
Positive BMI1 and KLF4 expression are associated with OS metastasis, and predicted a short survival time in patients. **(A)** Immunohistochemistry. The mouse OS cell xenografts and lung tissues were resected from the nude mice and subjected to tissue processing, embedding, sectioning, and immunohistochemistry (magnification, ×40). **(B)** The graph of the quantified data of immunohistochemical staining of BMI1 and KLF4. (**p < 0.01; ***p < 0.001; n = 3). **(C, D)** Kaplan-Meier analyses. BMI1 and KLF4 expression in OS was analyzed to predict the prognosis of OS patients by using Kaplan-Meier method on R2: Genomics Analysis and Visualization Platform. (*p* > 0.05; ** *p* < 0.01; **C, D**). The data were expressed as the mean ± SEM.

We tested the expression of BMI1 and KLF4 of primary and metastasis tumor in mouse OS xenografts, and the IHC results demonstrated that the expression of BMI1 and KLF4 was higher in metastasis tumor than that in primary (**p < 0.01; ***p < 0.001; **Figures 6A, B**). We further used the Kaplan-Meier methods to compute the survival analyses on R2: Genomics Analysis and Visualization Platform, and the results showed that positive BMI1 and KLF4 expression are associated worse survival rate in OS patients (*p* > 0.05; **p < 0.01; **Figures 6C, D**). This indicated that the BMI1 and KLF4 expression was valuable in predicting prognosis in OS.

### miR-135a Targeting of BMI1 and KLF4 in OS Cells

**Figure 7:**
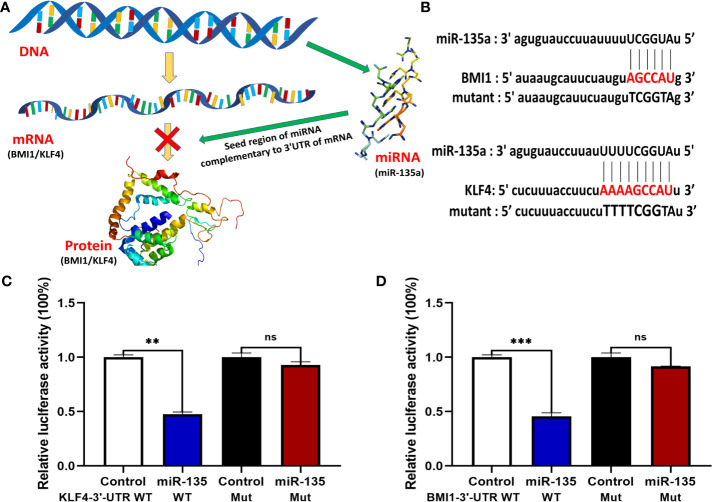
miR-135a targeting of BMI1 and KLF4 expression in OS cells. **(A)** A schematic diagram illustrated how miR-135a inhibited OS progression by targeting BMI1 and KLF4 pathway. **(B)** Bioinformatic analysis of miR-135a targeting of BMI1 and KLF4 3′-UTR. **(C, D)** Luciferase reporter assay. Saos-2 cells were grown and cotransfected with 10 nM of miR-135a mimic or control miR-NC and with 0.25 lg per/well of plasmids containing the 3′-UTR of BMI1 and KLF4 or their mutants using Lipofectamine 3000 for 2 days and then subjected to luciferase reporter assay. (ns *p* >0.05; **p < 0.01; ***p < 0.001; n = 3). The data were expressed as the mean ± SEM. ns, not significant.

We then used TargetScan and miRanda database to perform bioinformatics analysis and found potential binding sites for miR-135a (**Figures 7A, B**). We performed Luciferase assay to test the biological functions of predicted binding sites and the results revealed that upregulating miR-135a decreased the reporter activity of both the 3′ UTRs of WT BMI1 and KLF4 (**p < 0.01; ***p < 0.001; **Figures 7C, D**), whereas had no noticeable effect on the mutated reporter activity of BMI1 and KLF4 (*p* > 0.05; **Figures 7C, D**).

## Discussion

Mounting evidence supports the notion that aberrant BMI1 and KLF4 expression are responsible for tumor generation, metastasis, and treatment failure (10, 15, 17, 30). Accordingly, to achieve tumor eradication, new approaches capable of targeting these tumorigenic cores of cancers are needed. Many antioncogenes and drugs are reported to inhibit BMI1 or KLF4 expression in tumors, but few approaches can simultaneously suppress the expression of these two molecules, especially in OS (30–32). Consequently, targeting BMI1 and KLF4 simultaneously in OS may be the key for developing more effective treatments.

miRNAs are characterized as a group of endogenous noncoding RNAs that play key roles by binding to the protein-coding genes of mRNAs to direct their posttranscriptional repression and to regulate approximately 30%–50% of human gene expression (33). Previous studies indicated that miR-135 inhibits proliferation, invasion, and metastasis in several cancers, such as lung cancer, breast cancer, prostate cancer, tongue squamous cell carcinoma, and metastatic bone disease (34–38). Our current data on downregulation of miR-135a in OS are consistent with previous studies (39, 40). Although some of the studies found that miR-135 acts as an antioncogene in several cancers including OS (40), and this is the first report that miR-135a can inhibit OS development by targeting BMI1 and KLF4 *in vitro* and *in vivo*. Even though many studies showed the anti-tumor effect of miR-135a, there are many studies showing an oncogenic role for miR-135a (41). In hepatocellular carcinoma, for example, miR-135a promotes the migration and invasion of cancer cells by increasing the phosphorylation of AKT (42). Considering that different cancers may have completely different biological characteristics and genetic backgrounds, this seems to be explained by the unique role of miR-135a, because the expression levels of miR-135a target vary from different cancers (41). In the current study, we revealed that miR-135a was able to bind to BMI1 and KLF4 through the 3′-UTR and inhibit the translation or reduce BMI1/KLF4 mRNA expression in OS. This finding is consistent with previous studies reporting that miR-135 directly targets KLF4 (43) and its homolog KLF8 (38) and showing that miR-135 reduced cell proliferation (35), invasion, metastasis (36, 44) and increased sensitivity to chemotherapy (34) in a variety of cancer cells. Furthermore, the targeting of BMI1 or KLF4 has been demonstrated to benefit cancer patients or inhibit tumor progression in preclinical studies (17, 45–48). This finding is also consistent with our data that both BMI1 and KLF4 were inhibited by upregulation of miR-135a and that the development of OS was suppressed *in vitro* and *in vivo*. We found that in addition to KLF4 and BMI1, miR-135a also inhibited the expression of MMP2 and MMP9, as shown by Western blot, and inhibited MMP activation, as shown by the decrease in the intensity of MMPSense in FMT, which plays important roles in promoting tumor invasion and metastasis. These data are consistent with our previously published study (8). Meanwhile, Zheng et al. (38) showed that the decrease in MMP2 and MMP9 protein levels induced by DANCR silencing was partially increased by the miR-135a-5p inhibitor, which supports our result that miR-135a inhibits MMP2 and MMP9. Furthermore, we found that miR-135a exerted a better antineoplastic effect on tumor growth over time than PTC-209 and combination therapy in nude mouse xenograft model. These results indicated that miR-135a might act as a wide target to regulate multiple molecule functions in tumors. We will further investigate the underlying mechanisms of miR-135a as an antioncogene and its pros and cons in different development pathological stages of tumors in the future.

Our study does have some limitations. This is a small sample size study and lack the comprehensive investigation of the BMI1, KLF4, and MMP downstream signaling genes. In conclusion, this study revealed that abridged miR-135a expression in OS, while restoration of miR-135a suppressed tumor invasion and pulmonary metastasis by targeting BMI1 and KLF4 *in vitro* and *in vivo*. The present study extended previous findings of non-coding RNA regulation and provided insight into the miR-135a effects in OS progression. Future investigations of miR-135a as potential tumor suppressor could benefit patients to effectively block OS progression in clinic.

## Data Availability Statement

The datasets presented in this study can be found in online repositories. The names of the repository/repositories and accession number(s) can be found below: https://www.ncbi.nlm.nih.gov/, 406925.

## Ethics Statement

The animal study was reviewed and approved by the Ethics Committee of Shanxi Medical University (2017LL077).

## Author Contributions

XS, CLC, and ZL designed the study. WG and TR supervised the study. CLC, XM, CTC, and YJ performed the experiments. CLC and YZ analyzed the data. CLC and XS interpreted the results and wrote the manuscript. All authors contributed to the article and approved the submitted version.

## Funding

This work was supported by grants from the National Natural Science Foundation of China (nos. 81772867, 81572633), Natural Science Foundation of Beijing Municipality (no. 7182170), and Science and Technology Planning Project of Beijing (nos. Z1811000019118025, 2144000026).

## Conflict of Interest

The authors declare that the research was conducted in the absence of any commercial or financial relationships that could be construed as a potential conflict of interest.
