# Supplementary figures and images for: miR-135a Reduces Osteosarcoma Pulmonary Metastasis by Targeting Both BMI1 and KLF4
Source: Front Oncol. 2021 Mar 22;11:620295. doi: 10.3389/fonc.2021.620295 (PMC8019936; doi:10.3389/fonc.2021.620295)

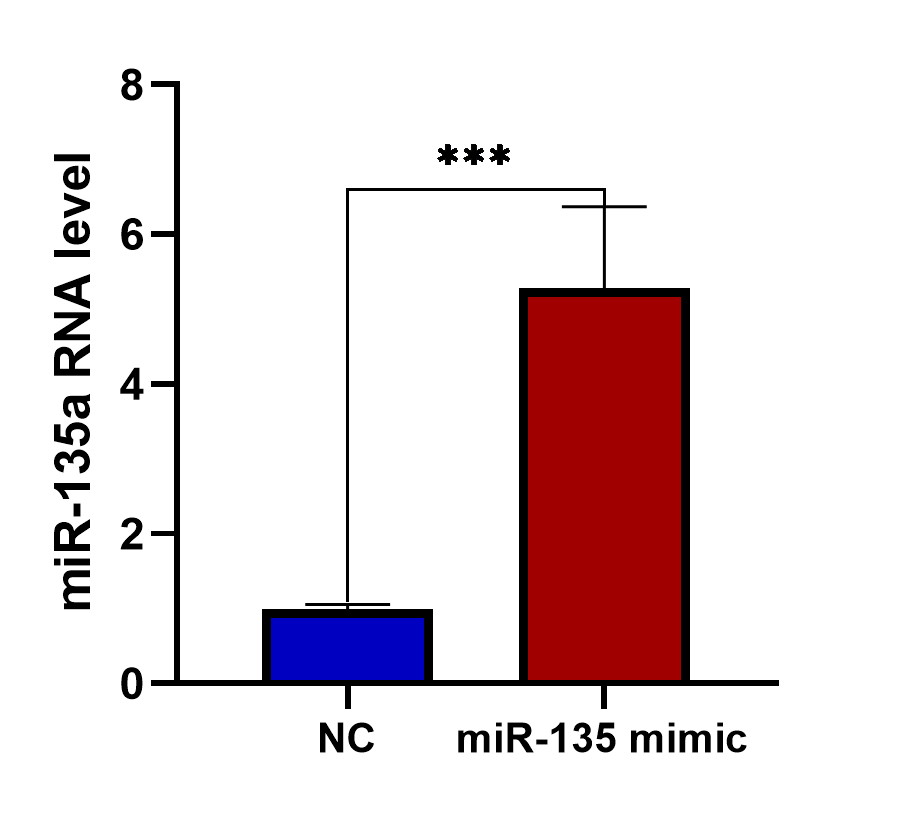

Supplement: Supplementary file 1 [file Image_1.tif]
